# A new dabco-templated metal sulfate: 1,4-diazo­niabicyclo­[2.2.2]octane hexa­aqua­cadmium bis­(sulfate)

**DOI:** 10.1107/S1600536812020089

**Published:** 2012-05-12

**Authors:** Bi-Qin Wang, Hai-Biao Yan, Ci-Jun Fang, Zhi Zhang

**Affiliations:** a School of Chemical and Environmental Engineering, Hubei University of Technology, Wuhan, Hubei 430068, People’s Republic of China; b School of Science, Hubei University of Technology, Wuhan, Hubei 430068, People’s Republic of China

## Abstract

The title double mol­ecular salt, (C_6_H_14_N_2_)[Cd(H_2_O)_6_](SO_4_)_2_, is an isostructure of its Mn and Co analogues. The Cd^II^ atom adopts a near-regular CdO_6_ octa­hedral coordination geometry. The crystal structure can be described as an alternation of cationic and anionic layers along [010], and numerous O—H⋯O and N—H⋯O hydrogen bonds are observed. No thermal anomalies corresponding to possible phase transitions were observed in DSC (differential scanning calorimetry) measurements and the 93 K structure is almost the same as the room-temperature structure.

## Related literature

For structural phase transitions of 1,4-diazoniabicyclo[2.2.2]octane-templated metal sulfates, see: Yahyaoui *et al.* (2007 ▶); Naili *et al.* (2006 ▶); Rekik *et al.* (2006 ▶); Zhang *et al.* (2009 ▶). For other related structures, see: Zhao *et al.* (2005 ▶); Rekik *et al.* (2007 ▶).
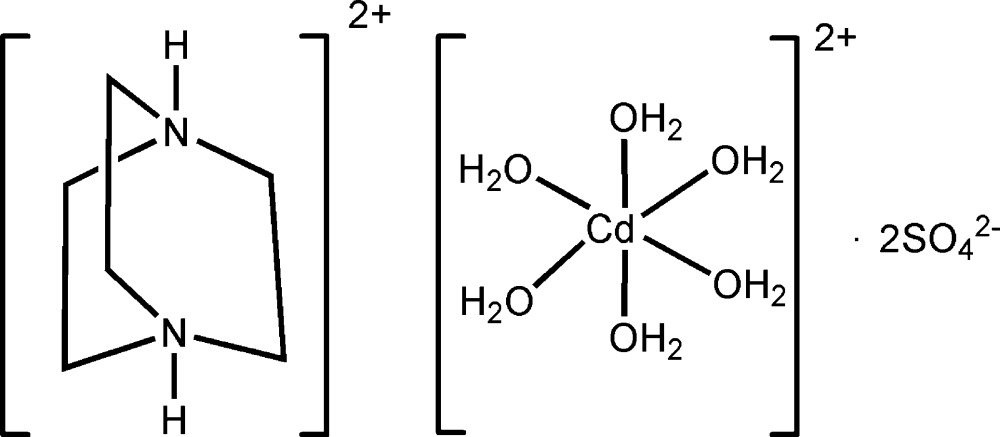


## Experimental

### 

#### Crystal data

(C_6_H_14_N_2_)[Cd(H_2_O)_6_](SO_4_)_2_
*M* *_r_* = 526.81
Monoclinic, 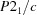

*a* = 12.201 (2) Å
*b* = 12.461 (3) Å
*c* = 12.377 (3) Å
β = 105.10 (3)°
*V* = 1816.8 (6) Å^3^
*Z* = 4
Mo *K*α radiation
μ = 1.50 mm^−1^
*T* = 298 K
0.45 × 0.40 × 0.35 mm

#### Data collection

Rigaku R-AXIS RAPID IP area-detector diffractometer
Absorption correction: multi-scan (*RAPID-AUTO*; Rigaku, 2000 ▶) *T*_min_ = 0.621, *T*_max_ = 0.818
17238 measured reflections
4123 independent reflections
3872 reflections with *I* > 2σ(*I*)
*R*_int_ = 0.034

#### Refinement

*R*[*F* ^2^ > 2σ(*F* ^2^)] = 0.026
*wR*(*F* ^2^) = 0.066
*S* = 1.11
4123 reflections
275 parameters
18 restraints
H atoms treated by a mixture of independent and constrained refinement
Δρ_max_ = 0.60 e Å^−3^
Δρ_min_ = −0.56 e Å^−3^

### 

Data collection: *RAPID-AUTO* (Rigaku, 2000 ▶); cell refinement: *RAPID-AUTO*; data reduction: *RAPID-AUTO* ; program(s) used to solve structure: *SHELXTL* (Sheldrick, 2008 ▶); program(s) used to refine structure: *SHELXTL*; molecular graphics: *SHELXTL* and *DIAMOND* (Brandenburg & Putz, 2005 ▶); software used to prepare material for publication: *SHELXTL*.

## Supplementary Material

Crystal structure: contains datablock(s) I, global. DOI: 10.1107/S1600536812020089/hb6712sup1.cif

Structure factors: contains datablock(s) I. DOI: 10.1107/S1600536812020089/hb6712Isup2.hkl

Additional supplementary materials:  crystallographic information; 3D view; checkCIF report

## Figures and Tables

**Table 1:** Selected bond lengths (Å)

Cd1—O4	2.2437 (16)
Cd1—O5	2.2514 (17)
Cd1—O2	2.2589 (17)
Cd1—O1	2.2629 (17)
Cd1—O3	2.3189 (18)
Cd1—O6	2.3534 (17)

**Table 2:** Hydrogen-bond geometry (Å, °)

*D*—H⋯*A*	*D*—H	H⋯*A*	*D*⋯*A*	*D*—H⋯*A*
O1—H1*A*⋯O9^i^	0.84 (2)	1.85 (2)	2.691 (2)	176 (3)
O1—H1*B*⋯O12	0.85 (2)	1.90 (2)	2.721 (2)	165 (3)
O2—H2*A*⋯O8	0.85 (2)	1.89 (2)	2.725 (2)	169 (3)
O2—H2*B*⋯O14^ii^	0.83 (2)	1.92 (2)	2.720 (3)	164 (3)
O3—H3*A*⋯O8^iii^	0.84 (2)	1.93 (2)	2.775 (3)	174 (3)
O3—H3*B*⋯O11^iv^	0.83 (2)	2.01 (2)	2.802 (3)	159 (3)
O4—H4*A*⋯O14^v^	0.85 (2)	1.82 (2)	2.666 (2)	176 (3)
O4—H4*B*⋯O13	0.84 (2)	1.85 (2)	2.680 (2)	171 (3)
O5—H5*A*⋯O10	0.83 (2)	1.92 (2)	2.741 (2)	171 (3)
O5—H5*B*⋯O9^iii^	0.82 (2)	1.87 (2)	2.686 (2)	172 (4)
O6—H6*A*⋯O12^v^	0.83 (2)	1.94 (2)	2.767 (2)	175 (3)
O6—H6*B*⋯O10^i^	0.84 (2)	2.07 (2)	2.902 (2)	175 (3)
N1—H1*E*⋯O11^vi^	0.91	1.94	2.749 (3)	147
N1—H1*E*⋯O12^vi^	0.91	2.36	3.140 (3)	144
N2—H2*E*⋯O7^iii^	0.91	1.78	2.671 (3)	164

Symmetry codes: (i) 
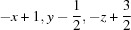
; (ii) 
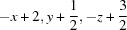
; (iii) 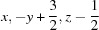; (iv) 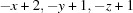; (v) 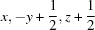; (vi) 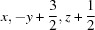.

**Fig. 1.**
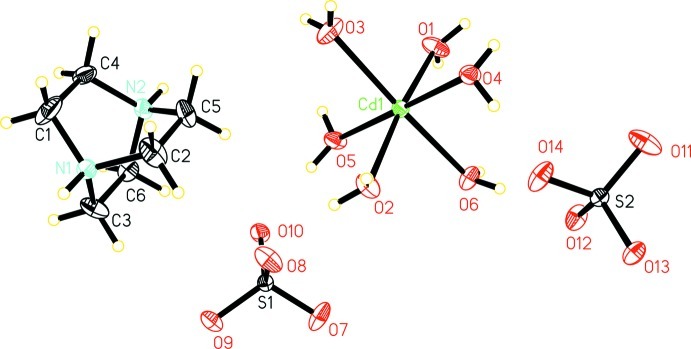
View of the asymmetric unit of I with displacement ellipsoids drawn at the 30% probability level.

**Fig. 2.**
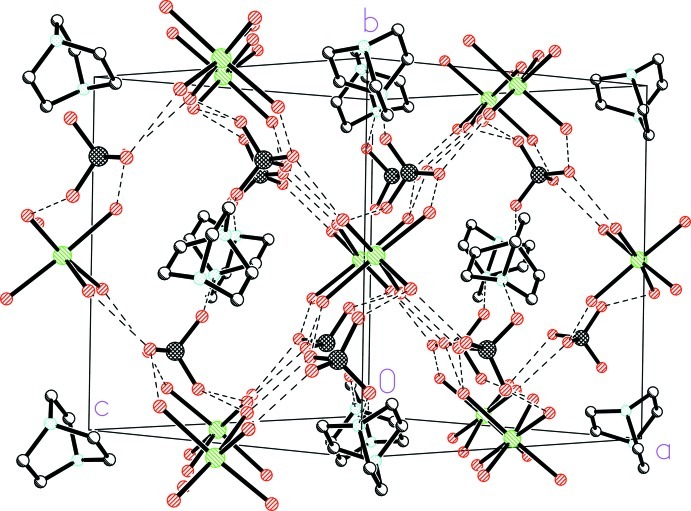
Dabcodiium templated three dimensional hydgrogen bonding network of I viewed down the [101] direction.

**Fig. 3.**
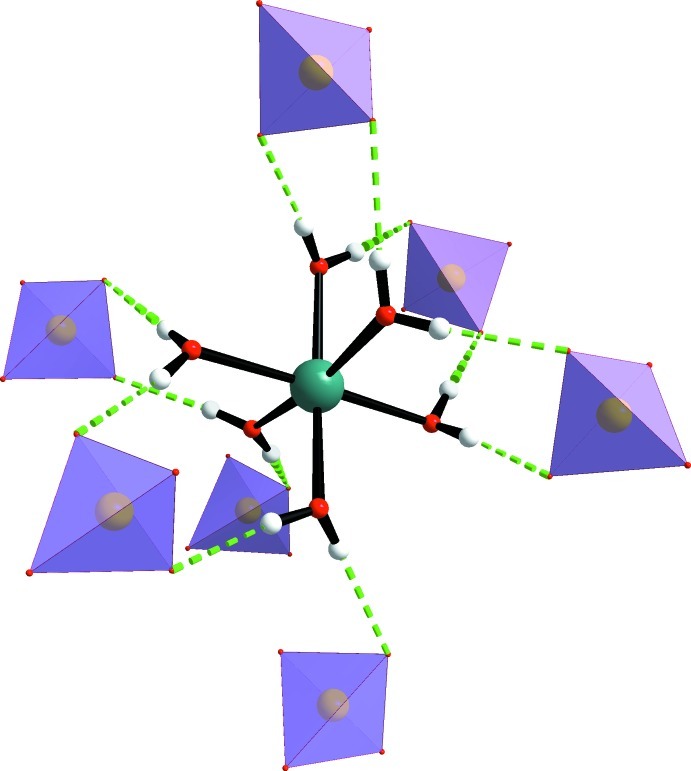
Neighboring sulfates in the environment of [Cd(H2O)6]2+ in I.

**Fig. 4.**
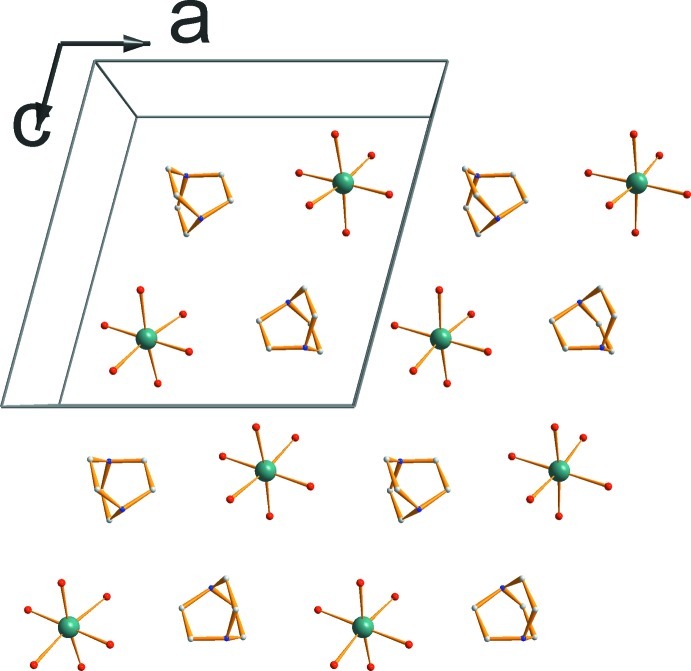
A cationic layer in the (ac) plane, showing the alteration of organic and inorganic cations.

**Fig. 5.**
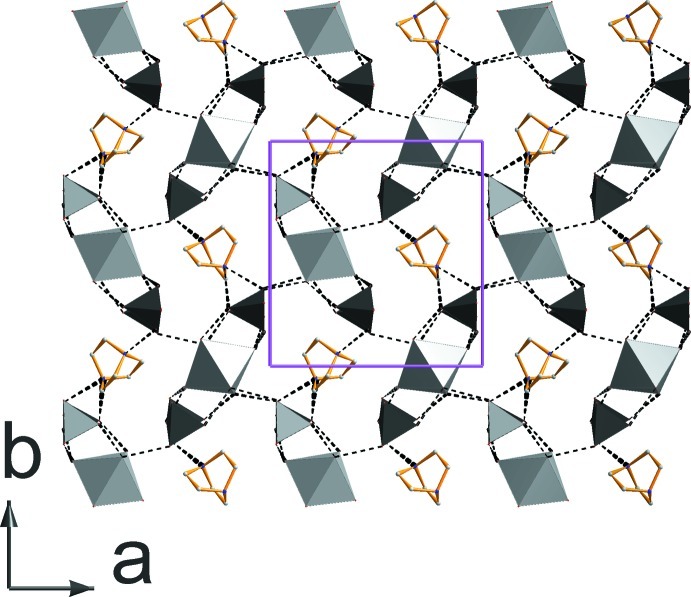
Projection of the crystal structure of I along the c axis, showing the alternation of the anionic and cationic layers along the b axis.

## supplementary crystallographic information

### Comment

1,4-diazoniabicyclo(2,2,2)octane (dabcodiium)-templated metal sulfates with general formula (C_6_H_14_N_2_)[*M*(H_2_O)_6_](SO_4_)_2_ (*M* = Mn, Ni, Fe, Co, Cu) are structurally interesting. These structures involve rich hydrogen bonding modes and thus several packing modes form among them (Yahyaoui *et al.*, 2007; Rekik *et al.*, 2006, 2007; Naili *et al.*, 2006; Zhao *et al.*, 2005). The related Fe, Ni, Cu compounds were found to undergo reversible phase transitions resulting from the ordering of the dabcodiium cations (Yahyaoui *et al.*, 2007; Naili *et al.*, 2006; Rekik *et al.*, 2006). Similarly, Dabcodiium hexaaquacopper(II) bis(selenate), (H_2_dabco)Cu(H_2_O_)6_(SeO_4_)_2_, as a new member of this series, was recently found to undergo a paraelectric-to-ferroelctric phase transition with striking dielectric response (Zhang *et al.*, 2009). It seems the structures and related structural phase transitions are sensitive to both the metal ions and the counterpart anions. So far, the metal ions in this series of compounds are limited to the first row transition metal. We herein report the structure of a new member of this series with the second transition metal ion, Dabcodiium hexaaquacadmium(II) bis(sulfate), (C_6_H_14_N2)[Cd(H_2_O)_6_](SO_4_)_2_ (I).

The crystal of I is monoclinic, spacegroup *P*2_1_/*c*, *a* = 12.201, *b* = 12.461, *c* = 12.377 Å, and β= 105.1°. Therefore, I was isostructural to the corresponding Mn or Co analogues (Rekik *et al.*, 2007; Zhao *et al.*, 2005). The structure consists of discrete [Cd(H_2_O)_6_]^2+^ octahedra, sulfate tetrahedra and dabcodiium cations linked together by a hydrogen bond network (Fig. 1 and 2, Table 1).

The [Cd(H_2_O)_6_]^2+^ octahedron is slightly irregular according to the Cd–OW distances and the OW–Cd–OW angles (Table 1). Each [Cd(H_2_O)_6_]^2+^ octahedron is surrounded by five sulfate groups H-bonded in a bidentate manner and two sulfate groups in a monodentate mode to the octahedron (Fig. 3).

The dabconium moieties can be viewed as template, and are stabilized in the hydrogen bonding network through N—H···O bonds (Fig. 2, Table 2).They occupy general positions and are fully ordered. The C–C and N–C distances range from 1.507 (3) to 1.512 (4) Å, from 1.481 (3) to 1.496 (3) Å respectively. As is the case in the Mn or Co isostructure. In the dabconium templated sulfates, phase transition was observed only for those with disordered dabcodiium cations. To confirm whether there is a phase transition, we performed DSC measurement. No thermal anomaly was observed in the temperature from 148 to below 373 K. Near 373 K the heat flow increases rapidly, indicating there is not a higher-temperature phase transition but decomposition. The structure determined at 93 K is shown to have the same structure at room temperature. The negative results prove the disordered dabcodiium play the key role in the phase transtions as well as packing modes of the structures.

Fig. 4 illustrates a cationic layer in the (*ac*) plane. Organic and inorganic cations alternate along the three crystallographic axes, so that each organic cation is surrounded by six inorganic cations in the structure, and *vice versa*. The organic moieties are stacked along [101] and [101] directions in the (*ac*) plane, so the inorganic cations are. The two independent sulfate anions have normal geometry, as seen in other dabcodiium-templated sulfates (Yahyaoui *et al.*, 2007; Rekik *et al.*, 2006, 2007; Naili *et al.*, 2006; Zhao *et al.*, 2005). As can be seen in Fig. 2 and 5, the sulfate anions play an important role in the structure connectivity. They are stacked in a manner that they form anionic layers parallel to the cationic ones parallel to the (*ac*) plane. Then cationic and anionic layers alternate along the *b* axis in a *ABAB* fashion and linked by N—H···O and OW—H···O. (Fig. 5). The crystal structure is then described as an alternation of cationic and anionic layers along [010].

### Experimental

An aqueous solution of dabcodiium sulfate was prepared by neutralization of dabco with equimolar amount of sulfuric acid in water. To this solution, an aqueous solution containing equimolar amount of CdSO_4_ was added. The resulting solution was allowed to evaporate at room temperature and colourless blocks of (I) were obtained after two weeks, Yield: 70%.

### Refinement

All H atoms were found in the difference maps. Those from coordinated water molecules was refined isotropically. The bond distances of O—H and distance between two H atoms from each water molecules was restrained to be 0.85 and 1.37 Å with the default deviation respectively. However, those bonded to C and N atoms were placed at ideal positions and refined using a 'riding' model with *U*_iso_ = 1.2 *U*_eq_ (C or N).

### Crystal data

**Table:**

(C_6_H_14_N_2_)[Cd(H_2_O)_6_](SO_4_)_2_	*F*(000) = 1072
*M**_r_* = 526.81	*D*_x_ = 1.926 Mg m^−^^3^
Monoclinic, *P*2_1_/*c*	Mo *K*α radiation, λ = 0.71073 Å
*a* = 12.201 (2) Å	Cell parameters from 14955 reflections
*b* = 12.461 (3) Å	θ = 3.2–27.4°
*c* = 12.377 (3) Å	µ = 1.50 mm^−^^1^
β = 105.10 (3)°	*T* = 298 K
*V* = 1816.8 (6) Å^3^	Block, colourless
*Z* = 4	0.45 × 0.40 × 0.35 mm

### Data collection

**Table:**

Rigaku R-AXIS RAPID IP area-detector diffractometer	4123 independent reflections
Radiation source: Rotating anode target	3872 reflections with *I* > 2σ(*I*)
Graphite monochromator	*R*_int_ = 0.034
ω scans	θ_max_ = 27.4°, θ_min_ = 3.2°
Absorption correction: multi-scan (*RAPID-AUTO*; Rigaku, 2000)	*h* = −15→15
*T*_min_ = 0.621, *T*_max_ = 0.818	*k* = −16→16
17238 measured reflections	*l* = −16→16

### Refinement

**Table:**

Refinement on *F*^2^	Secondary atom site location: difference Fourier map
Least-squares matrix: full	Hydrogen site location: inferred from neighbouring sites
*R*[*F*^2^ > 2σ(*F*^2^)] = 0.026	H atoms treated by a mixture of independent and constrained refinement
*wR*(*F*^2^) = 0.066	*w* = 1/[σ^2^(*F*_o_^2^) + (0.0296*P*)^2^ + 1.2094*P*] where *P* = (*F*_o_^2^ + 2*F*_c_^2^)/3
*S* = 1.11	(Δ/σ)_max_ = 0.001
4123 reflections	Δρ_max_ = 0.60 e Å^−^^3^
275 parameters	Δρ_min_ = −0.56 e Å^−^^3^
18 restraints	Extinction correction: *SHELXTL* (Sheldrick, 2008), Fc^*^=kFc[1+0.001xFc^2^λ^3^/sin(2θ)]^-1/4^
Primary atom site location: structure-invariant direct methods	Extinction coefficient: 0.0073 (4)

### Special details

**Table:**

Geometry. All e.s.d.'s (except the e.s.d. in the dihedral angle between two l.s. planes) are estimated using the full covariance matrix. The cell e.s.d.'s are taken into account individually in the estimation of e.s.d.'s in distances, angles and torsion angles; correlations between e.s.d.'s in cell parameters are only used when they are defined by crystal symmetry. An approximate (isotropic) treatment of cell e.s.d.'s is used for estimating e.s.d.'s involving l.s. planes.
Refinement. Refinement of *F*^2^ against ALL reflections. The weighted *R*-factor *wR* and goodness of fit *S* are based on *F*^2^, conventional *R*-factors *R* are based on *F*, with *F* set to zero for negative *F*^2^. The threshold expression of *F*^2^ > σ(*F*^2^) is used only for calculating *R*-factors(gt) *etc*. and is not relevant to the choice of reflections for refinement. *R*-factors based on *F*^2^ are statistically about twice as large as those based on *F*, and *R*-factors based on ALL data will be even larger.

### Fractional atomic coordinates and isotropic or equivalent isotropic displacement parameters (Å^2^)

**Table:**

	*x*	*y*	*z*	*U*_iso_*/*U*_eq_	
Cd1	0.762711 (12)	0.506501 (11)	0.729425 (12)	0.02484 (7)	
O1	0.69454 (15)	0.40460 (16)	0.57520 (15)	0.0434 (4)	
H1A	0.6259 (15)	0.386 (2)	0.564 (3)	0.058 (10)*	
H1B	0.729 (2)	0.3523 (19)	0.556 (2)	0.045 (8)*	
O2	0.81582 (14)	0.59718 (14)	0.89279 (15)	0.0373 (4)	
H2A	0.775 (2)	0.651 (2)	0.899 (3)	0.057 (10)*	
H2B	0.8838 (14)	0.613 (2)	0.917 (2)	0.044 (8)*	
O3	0.83071 (16)	0.62381 (18)	0.61663 (18)	0.0520 (5)	
H3A	0.786 (2)	0.654 (3)	0.561 (2)	0.059 (10)*	
H3B	0.8981 (15)	0.630 (3)	0.616 (3)	0.065 (10)*	
O4	0.93016 (14)	0.42094 (14)	0.77884 (13)	0.0335 (3)	
H4A	0.943 (3)	0.394 (2)	0.8444 (15)	0.054 (9)*	
H4B	0.937 (3)	0.373 (2)	0.734 (2)	0.066 (11)*	
O5	0.59756 (15)	0.59730 (15)	0.68221 (14)	0.0400 (4)	
H5A	0.579 (3)	0.646 (2)	0.719 (2)	0.061 (10)*	
H5B	0.583 (3)	0.610 (3)	0.6150 (15)	0.076 (12)*	
O6	0.67448 (15)	0.38020 (13)	0.82049 (14)	0.0367 (4)	
H6A	0.717 (2)	0.341 (2)	0.868 (2)	0.048 (8)*	
H6B	0.6206 (18)	0.344 (2)	0.782 (2)	0.042 (8)*	
S1	0.56662 (4)	0.75687 (4)	0.92161 (4)	0.02197 (11)	
O7	0.53791 (18)	0.66368 (15)	0.98053 (16)	0.0516 (5)	
O8	0.69086 (14)	0.76330 (15)	0.94054 (16)	0.0453 (4)	
O9	0.52740 (14)	0.85701 (13)	0.96289 (13)	0.0371 (4)	
O10	0.51233 (14)	0.74705 (12)	0.80082 (12)	0.0339 (3)	
S2	0.93025 (4)	0.25840 (4)	0.51135 (4)	0.02264 (11)	
O11	0.96652 (16)	0.34160 (17)	0.44445 (17)	0.0538 (5)	
O12	0.80398 (13)	0.25778 (14)	0.47823 (15)	0.0391 (4)	
O13	0.97215 (15)	0.28065 (14)	0.63040 (13)	0.0377 (4)	
O14	0.97126 (16)	0.15212 (15)	0.48799 (14)	0.0434 (4)	
N1	0.78520 (16)	1.05733 (14)	0.80199 (15)	0.0288 (4)	
H1E	0.8228	1.0984	0.8608	0.035*	
N2	0.68305 (16)	0.94584 (14)	0.64165 (15)	0.0305 (4)	
H2E	0.6453	0.9047	0.5830	0.037*	
C1	0.7918 (3)	1.1099 (3)	0.6964 (2)	0.0670 (10)	
H1C	0.8706	1.1173	0.6951	0.080*	
H1D	0.7586	1.1811	0.6918	0.080*	
C2	0.8375 (2)	0.9493 (2)	0.8128 (2)	0.0515 (7)	
H2C	0.8310	0.9157	0.8816	0.062*	
H2D	0.9174	0.9552	0.8155	0.062*	
C3	0.6649 (2)	1.0471 (2)	0.8051 (2)	0.0445 (6)	
H3C	0.6304	1.1176	0.8009	0.053*	
H3D	0.6608	1.0138	0.8748	0.053*	
C4	0.7286 (2)	1.0432 (2)	0.59765 (19)	0.0405 (5)	
H4C	0.6669	1.0846	0.5509	0.049*	
H4D	0.7795	1.0222	0.5528	0.049*	
C5	0.7775 (2)	0.8816 (2)	0.7136 (2)	0.0449 (6)	
H5C	0.8305	0.8606	0.6710	0.054*	
H5D	0.7476	0.8170	0.7391	0.054*	
C6	0.60209 (19)	0.97960 (19)	0.7075 (2)	0.0336 (5)	
H6C	0.5703	0.9168	0.7344	0.040*	
H6D	0.5403	1.0207	0.6604	0.040*	

### Atomic displacement parameters (Å^2^)

**Table:**

	*U*^11^	*U*^22^	*U*^33^	*U*^12^	*U*^13^	*U*^23^
Cd1	0.02507 (10)	0.02523 (10)	0.02356 (10)	0.00126 (5)	0.00517 (6)	0.00051 (5)
O1	0.0283 (9)	0.0601 (12)	0.0413 (9)	−0.0027 (8)	0.0081 (7)	−0.0239 (9)
O2	0.0262 (8)	0.0391 (9)	0.0420 (9)	−0.0011 (7)	0.0004 (7)	−0.0159 (7)
O3	0.0330 (10)	0.0659 (13)	0.0580 (12)	0.0044 (9)	0.0138 (9)	0.0350 (10)
O4	0.0336 (8)	0.0401 (9)	0.0272 (8)	0.0088 (7)	0.0087 (7)	0.0033 (7)
O5	0.0422 (9)	0.0534 (11)	0.0230 (8)	0.0214 (8)	0.0057 (7)	0.0019 (7)
O6	0.0336 (9)	0.0359 (9)	0.0349 (9)	−0.0072 (7)	−0.0011 (7)	0.0104 (7)
S1	0.0208 (2)	0.0220 (2)	0.0202 (2)	0.00136 (17)	0.00037 (17)	0.00003 (16)
O7	0.0549 (11)	0.0445 (10)	0.0450 (10)	−0.0107 (9)	−0.0054 (9)	0.0236 (8)
O8	0.0232 (8)	0.0536 (11)	0.0573 (11)	−0.0003 (7)	0.0072 (7)	−0.0234 (9)
O9	0.0352 (8)	0.0403 (9)	0.0318 (8)	0.0115 (7)	0.0016 (7)	−0.0109 (7)
O10	0.0420 (9)	0.0338 (8)	0.0209 (7)	0.0046 (7)	−0.0008 (6)	−0.0040 (6)
S2	0.0215 (2)	0.0245 (2)	0.0205 (2)	0.00259 (17)	0.00292 (17)	0.00059 (17)
O11	0.0407 (10)	0.0647 (13)	0.0575 (12)	0.0032 (9)	0.0158 (9)	0.0362 (10)
O12	0.0220 (7)	0.0454 (9)	0.0463 (9)	−0.0003 (7)	0.0025 (7)	−0.0153 (8)
O13	0.0416 (9)	0.0423 (9)	0.0256 (8)	−0.0029 (7)	0.0024 (7)	−0.0100 (7)
O14	0.0445 (9)	0.0406 (10)	0.0348 (9)	0.0198 (8)	−0.0079 (7)	−0.0123 (7)
N1	0.0310 (9)	0.0284 (9)	0.0242 (8)	−0.0067 (7)	0.0023 (7)	−0.0048 (7)
N2	0.0358 (10)	0.0271 (9)	0.0247 (8)	−0.0043 (7)	0.0006 (7)	−0.0075 (7)
C1	0.102 (3)	0.0605 (19)	0.0367 (14)	−0.0519 (19)	0.0150 (16)	0.0042 (13)
C2	0.0420 (14)	0.0556 (17)	0.0457 (15)	0.0237 (13)	−0.0085 (12)	−0.0069 (13)
C3	0.0315 (12)	0.0614 (17)	0.0408 (13)	0.0087 (11)	0.0100 (10)	−0.0180 (12)
C4	0.0510 (15)	0.0476 (14)	0.0257 (11)	−0.0074 (12)	0.0149 (10)	0.0040 (10)
C5	0.0470 (14)	0.0302 (12)	0.0533 (15)	0.0119 (10)	0.0055 (12)	−0.0065 (11)
C6	0.0239 (10)	0.0381 (11)	0.0365 (12)	−0.0055 (9)	0.0037 (9)	0.0018 (9)

### Geometric parameters (Å, º)

**Table:**

Cd1—O4	2.2437 (16)	S2—O12	1.4875 (16)
Cd1—O5	2.2514 (17)	N1—C2	1.481 (3)
Cd1—O2	2.2589 (17)	N1—C1	1.483 (3)
Cd1—O1	2.2629 (17)	N1—C3	1.484 (3)
Cd1—O3	2.3189 (18)	N1—H1E	0.9100
Cd1—O6	2.3534 (17)	N2—C5	1.493 (3)
O1—H1A	0.844 (17)	N2—C4	1.496 (3)
O1—H1B	0.847 (16)	N2—C6	1.496 (3)
O2—H2A	0.848 (17)	N2—H2E	0.9100
O2—H2B	0.827 (17)	C1—C4	1.512 (4)
O3—H3A	0.844 (17)	C1—H1C	0.9700
O3—H3B	0.829 (17)	C1—H1D	0.9700
O4—H4A	0.851 (17)	C2—C5	1.512 (4)
O4—H4B	0.838 (17)	C2—H2C	0.9700
O5—H5A	0.825 (17)	C2—H2D	0.9700
O5—H5B	0.820 (17)	C3—C6	1.507 (3)
O6—H6A	0.833 (17)	C3—H3C	0.9700
O6—H6B	0.839 (16)	C3—H3D	0.9700
S1—O7	1.4615 (18)	C4—H4C	0.9700
S1—O10	1.4740 (16)	C4—H4D	0.9700
S1—O8	1.4746 (17)	C5—H5C	0.9700
S1—O9	1.4747 (16)	C5—H5D	0.9700
S2—O13	1.4550 (16)	C6—H6C	0.9700
S2—O11	1.4654 (18)	C6—H6D	0.9700
S2—O14	1.4707 (17)		
			
O4—Cd1—O5	178.14 (7)	C1—N1—C3	110.0 (2)
O4—Cd1—O2	88.00 (7)	C2—N1—H1E	108.9
O5—Cd1—O2	90.55 (7)	C1—N1—H1E	108.9
O4—Cd1—O1	94.12 (7)	C3—N1—H1E	108.9
O5—Cd1—O1	87.45 (7)	C5—N2—C4	110.5 (2)
O2—Cd1—O1	172.97 (7)	C5—N2—C6	110.05 (19)
O4—Cd1—O3	91.17 (7)	C4—N2—C6	109.35 (18)
O5—Cd1—O3	87.90 (7)	C5—N2—H2E	109.0
O2—Cd1—O3	99.17 (8)	C4—N2—H2E	109.0
O1—Cd1—O3	87.50 (8)	C6—N2—H2E	109.0
O4—Cd1—O6	92.83 (6)	N1—C1—C4	109.6 (2)
O5—Cd1—O6	88.30 (7)	N1—C1—H1C	109.7
O2—Cd1—O6	88.06 (7)	C4—C1—H1C	109.7
O1—Cd1—O6	85.14 (7)	N1—C1—H1D	109.7
O3—Cd1—O6	171.86 (7)	C4—C1—H1D	109.7
Cd1—O1—H1A	115 (2)	H1C—C1—H1D	108.2
Cd1—O1—H1B	125 (2)	N1—C2—C5	109.16 (19)
H1A—O1—H1B	107 (2)	N1—C2—H2C	109.8
Cd1—O2—H2A	116 (2)	C5—C2—H2C	109.8
Cd1—O2—H2B	118 (2)	N1—C2—H2D	109.8
H2A—O2—H2B	110 (2)	C5—C2—H2D	109.8
Cd1—O3—H3A	121 (2)	H2C—C2—H2D	108.3
Cd1—O3—H3B	125 (2)	N1—C3—C6	109.07 (18)
H3A—O3—H3B	112 (2)	N1—C3—H3C	109.9
Cd1—O4—H4A	112 (2)	C6—C3—H3C	109.9
Cd1—O4—H4B	114 (2)	N1—C3—H3D	109.9
H4A—O4—H4B	109 (2)	C6—C3—H3D	109.9
Cd1—O5—H5A	126 (2)	H3C—C3—H3D	108.3
Cd1—O5—H5B	108 (3)	N2—C4—C1	108.13 (18)
H5A—O5—H5B	114 (3)	N2—C4—H4C	110.1
Cd1—O6—H6A	117 (2)	C1—C4—H4C	110.1
Cd1—O6—H6B	118.2 (19)	N2—C4—H4D	110.1
H6A—O6—H6B	110 (2)	C1—C4—H4D	110.1
O7—S1—O10	109.72 (11)	H4C—C4—H4D	108.4
O7—S1—O8	109.51 (12)	N2—C5—C2	108.66 (19)
O10—S1—O8	109.72 (11)	N2—C5—H5C	110.0
O7—S1—O9	110.97 (12)	C2—C5—H5C	110.0
O10—S1—O9	108.73 (9)	N2—C5—H5D	110.0
O8—S1—O9	108.17 (10)	C2—C5—H5D	110.0
O13—S2—O11	111.06 (12)	H5C—C5—H5D	108.3
O13—S2—O14	108.84 (10)	N2—C6—C3	108.90 (18)
O11—S2—O14	110.96 (12)	N2—C6—H6C	109.9
O13—S2—O12	110.22 (10)	C3—C6—H6C	109.9
O11—S2—O12	106.95 (11)	N2—C6—H6D	109.9
O14—S2—O12	108.79 (10)	C3—C6—H6D	109.9
C2—N1—C1	111.1 (2)	H6C—C6—H6D	108.3
C2—N1—C3	109.0 (2)		

### Hydrogen-bond geometry (Å, º)

**Table:**

*D*—H···*A*	*D*—H	H···*A*	*D*···*A*	*D*—H···*A*
O1—H1*A*···O9^i^	0.84 (2)	1.85 (2)	2.691 (2)	176 (3)
O1—H1*B*···O12	0.85 (2)	1.90 (2)	2.721 (2)	165 (3)
O2—H2*A*···O8	0.85 (2)	1.89 (2)	2.725 (2)	169 (3)
O2—H2*B*···O14^ii^	0.83 (2)	1.92 (2)	2.720 (3)	164 (3)
O3—H3*A*···O8^iii^	0.84 (2)	1.93 (2)	2.775 (3)	174 (3)
O3—H3*B*···O11^iv^	0.83 (2)	2.01 (2)	2.802 (3)	159 (3)
O4—H4*A*···O14^v^	0.85 (2)	1.82 (2)	2.666 (2)	176 (3)
O4—H4*B*···O13	0.84 (2)	1.85 (2)	2.680 (2)	171 (3)
O5—H5*A*···O10	0.83 (2)	1.92 (2)	2.741 (2)	171 (3)
O5—H5*B*···O9^iii^	0.82 (2)	1.87 (2)	2.686 (2)	172 (4)
O6—H6*A*···O12^v^	0.83 (2)	1.94 (2)	2.767 (2)	175 (3)
O6—H6*B*···O10^i^	0.84 (2)	2.07 (2)	2.902 (2)	175 (3)
N1—H1*E*···O11^vi^	0.91	1.94	2.749 (3)	147
N1—H1*E*···O12^vi^	0.91	2.36	3.140 (3)	144
N2—H2*E*···O7^iii^	0.91	1.78	2.671 (3)	164

Symmetry codes: (i) −*x*+1, *y*−1/2, −*z*+3/2; (ii) −*x*+2, *y*+1/2, −*z*+3/2; (iii) *x*, −*y*+3/2, *z*−1/2; (iv) −*x*+2, −*y*+1, −*z*+1; (v) *x*, −*y*+1/2, *z*+1/2; (vi) *x*, −*y*+3/2, *z*+1/2.
